# Multiple Leptin Signalling Pathways in the Control of Metabolism and Fertility: A Means to Different Ends?

**DOI:** 10.3390/ijms22179210

**Published:** 2021-08-26

**Authors:** Maggie C. Evans, Rebecca A. Lord, Greg M. Anderson

**Affiliations:** Centre for Neuroendocrinology, Department of Anatomy, University of Otago, Dunedin 9016, New Zealand; lorre527@student.otago.ac.nz (R.A.L.); greg.anderson@otago.ac.nz (G.M.A.)

**Keywords:** leptin, signalling pathways, fertility, metabolism, leptin resistance, STAT3, PI3K, Akt, CRTC

## Abstract

The adipocyte-derived ‘satiety promoting’ hormone, leptin, has been identified as a key central regulator of body weight and fertility, such that its absence leads to obesity and infertility. Plasma leptin levels reflect body adiposity, and therefore act as an ‘adipostat’, whereby low leptin levels reflect a state of low body adiposity (under-nutrition/starvation) and elevated leptin levels reflect a state of high body adiposity (over-nutrition/obesity). While genetic leptin deficiency is rare, obesity-related leptin resistance is becoming increasingly common. In the absence of adequate leptin sensitivity, leptin is unable to exert its ‘anti-obesity’ effects, thereby exacerbating obesity. Furthermore, extreme leptin resistance and consequent low or absent leptin signalling resembles a state of starvation and can thus lead to infertility. However, leptin resistance occurs on a spectrum, and it is possible to be resistant to leptin’s metabolic effects while retaining leptin’s permissive effects on fertility. This may be because leptin exerts its modulatory effects on energy homeostasis and reproductive function through discrete intracellular signalling pathways, and these pathways are differentially affected by the molecules that promote leptin resistance. This review discusses the potential mechanisms that enable leptin to exert differential control over metabolic and reproductive function in the contexts of healthy leptin signalling and of diet-induced leptin resistance.

## 1. Introduction

Leptin is the adipocyte-derived ‘anti-obesity’ or ‘starvation response’ hormone (depending which extreme of its circulating concentration range is being focused on) [1]. It is the product of the obese (*OB*) gene and plays a critical role in the maintenance of both metabolic and reproductive function, such that genetic leptin deficiency is associated with obesity, type II diabetes, and infertility in laboratory rodents [2,3] as well as humans [4,5]. Leptin is secreted peripherally into the bloodstream but exerts its critical regulatory effects on metabolic and reproductive function primarily by targeting long-form leptin receptor (LepRb)-expressing neurons in the brain [6,7]. Under normal conditions, leptin circulates in the blood and cerebrospinal fluid at concentrations that are proportional to white adipose tissue stores [8,9], thereby relaying information about peripheral energy storage status to the brain. During times of energy scarcity, which are inopportune times to reproduce, low circulating leptin levels mediate the suppression of reproduction function [10]. Circulating leptin levels also reflect circadian fluctuations as well as nutritional state [11,12], thus enabling leptin to play an important role in the control of feeding circuits (as reviewed in [13]), such that low circulating leptin levels drive food-seeking behaviour [14,15]. However, when adiposity increases and leptin levels become chronically elevated, leptin resistance eventually develops [16]. In the absence of adequate leptin sensitivity, feeding circuits and other leptin-regulated processes become dysregulated. While human leptin resistance is most commonly observed in modern times when energy-dense food is always readily available, a potential evolutionary significance of it may be to temporarily facilitate excessive nutrient intake during times of plenty so that reserves are built up in anticipation of the periods of food insecurity to come [1].

Leptin signalling is negatively regulated by intracellular proteins to prevent overactivation of LepRb pathways. At least some of these negative regulators of leptin are target genes of leptin-LepRb signalling, including suppressor of cytokine signalling 3 (SCOS3) and protein tyrosine phosphatase 1B (PTP1B), and thus become increasingly active as leptin levels increase [17], suppressing leptin sensitivity in a negative feedback manner. When circulating leptin levels are chronically elevated, as is the case in obesity, overactivation of leptin’s negative regulators can lead to leptin resistance [17,18]. Some leptin negative regulators are upregulated in response to endoplasmic reticulum stress rather than chronic leptin signalling [19], a situation that is also linked to obesity. In the absence of sufficient leptin sensitivity, leptin is unable to exert its satiety effects, which further promotes energy intake, adiposity, and hyperleptinemia. As one might expect in response to widespread leptin resistance, which resembles a situation of low leptin signalling (i.e., energy scarcity), diet-induced reproductive dysfunction can eventually develop [20]. However, while obesity-related infertility is becoming increasingly common [21], it is nevertheless possible to exhibit resistance to leptin’s ‘anti-obesity’ effects while remaining fertile [22]. For example, the fact that central leptin resistance leads to dysregulation of feeding much more quickly than dysregulation of fertility [23] presumably allows for excessive late summer and autumn feeding and fat accumulation without disruption of fertility in seasonal animals that mate at this time. Improving our understanding of how the development of leptin resistance differentially impacts metabolic and reproductive function is therefore an active area of research.

## 2. Neuronal Targets of Leptin Receptor Signalling in the Brain

One way whereby leptin exerts its differential modulatory effects on metabolic and reproductive function is by signalling via different LepR-expressing neuronal populations. Over the past two decades, targeted gene-deletion and gene-rescue experiments have made it possible to test both the functional requirement and sufficiency, respectively, of leptin signalling via discrete neuronal populations in the control of metabolic and/or reproductive function. Importantly, leptin does not directly interact with the gonadotrophin-releasing hormone (GnRH) neurons that control the hypothalamo–pituitary–gonadal axis [7], so it presumably acts via afferent inputs to GnRH neurons. As we have recently reviewed [24], LepRb deletion from many different discrete neuronal populations, including neurons expressing steroidogenic factor 1 (SF1) [25], agouti-related peptide (AgRP)/neuropeptide Y (NPY) and pro-opiomelanocortin (POMC) neurons [26], results in an overweight/obese phenotype, whereas reproductive dysfunction is only observed in response to more widespread LepRb deletion, such as from all forebrain neurons [7] and all GABAergic neurons [27]. Minor reproductive dysfunction (i.e., delayed female puberty) occurs in agouti-related peptide (AgRP) neuronal LepRb knockout mice [28]. In contrast, fertility, but not energy homeostasis, can be completely rescued in LepRb-deficient mice by restoring LepRbs exclusively to agouti-related peptide AgRP neurons [28]. A relatively small portion of kisspeptin neurons, which provide the most potent stimulatory input to GnRH neurons, are leptin-responsive. Leptin treatment can increase Kiss1 mRNA levels, and leptin-deficient mice show reduced Kiss1 expression in the hypothalamus [29]. However, deletion of LepRb from kisspeptin neurons does not alter reproductive function or body weight [30] and highlighting leptin signalling in kisspeptin neurons is not critically required for normal body weight or HPG function. These data highlight the evolutionary importance of safeguarding feeding behaviour and fertility, which are both critical for species survival, via complex, potentially redundant neuronal circuits. 

Even though leptin targets different neuronal populations to differentially modulate metabolic and reproductive function, there is abundant overlap in the leptin-targeted neurons in metabolic vs. reproductive control. While still not fully understood, it appears leptin can exert discrete effects on metabolic vs. reproductive control even when acting on the same neurons because, upon leptin-LepRb binding, different intracellular signalling pathways may be activated to carry out leptin’s metabolic vs. reproductive effects. It is therefore potentially possible for one leptin-targeted pathway to remain sensitive while another develops resistance, even within the same neuron. While LepRb gene-deletion studies are able to model a complete lack of leptin signalling on a given cell type, they are unable to model the progression and nuance of diet-induced cellular leptin resistance. The focus of this review will therefore be on impairments in leptin-activated cell signalling pathways and their respective impacts on metabolic vs. reproductive function (see Table 1 for a listing of experiments in which these have been addressed by deleting LepRb signalling molecules either globally or from specific cell types). We will also address the currently identified mechanism(s) of diet-induced leptin resistance and how they differentially contribute to obesity and infertility.

## 3. Leptin Receptor Signalling Pathways

**Figure 1:**
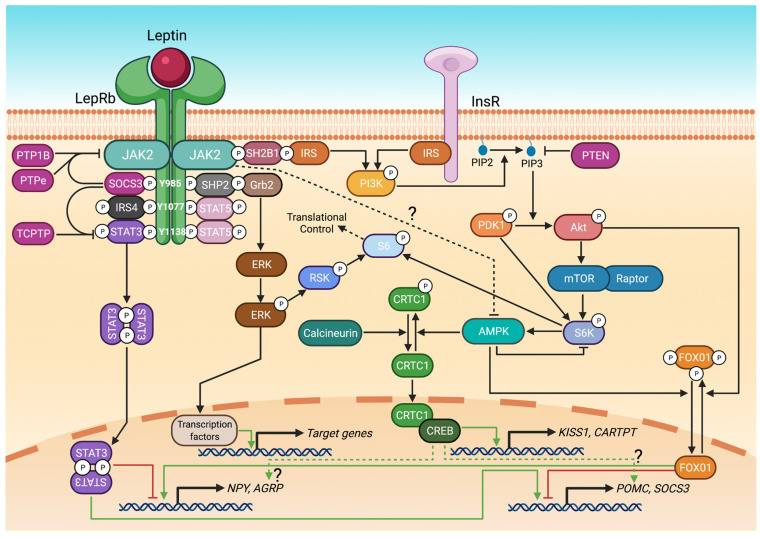
Summary of the major signalling pathways used by the LepRb in the hypothalamus to regulate metabolic and reproductive function. See text for explanatory details. AGRP, agouti-related peptide; Akt is also known as protein kinase B; AMPK, adenosine monophosphate-activated protein kinase; CARTPT, cocaine- and amphetamine-regulated transcript prepropeptide; CREB, cAMP response element binding protein; CRTC, CREB-regulated transcriptional coactivator; ERK, extracellular signal-regulated kinase; FOXO1, forkhead box protein O1; Grb2, growth factor receptor-bound protein 2; InsR, insulin receptor; IRS, insulin receptor substrate; IRS4, insulin receptor substrate 4; JAK2, Janus kinase 2; KISS1, kisspeptin gene; LepRb, long form of the leptin receptor; mTOR, mammalian target of rapamycin; NPY, neuropeptide Y; PDK1, phosphatidylinositol-dependent protein kinase 1; PI3K, phosphoinositide 3-kinase; PIP2, phosphatidylinositol 4,5-biphosphate; PIP3, phosphatidylinositol 3,4,5-triphosphate; POMC, pro-opiomelanocortin; PTEN, phosphatase and tensin homolog deleted on chromosome 10; PTP1B, protein tyrosine phosphatase 1B; PTPe, protein tyrosine phosphatase epsilon; RSK, p90 ribosomal S6 kinase; S6, ribosomal protein S6; S6K, p70 S6 kinase; SHP2, Src homology-2 domain-containing protein tyrosine phosphatase-2; SH2B1, Src homology-2 B adaptor protein 1; SOCS3, suppressor of cytokine signalling 3; STAT3 and STAT5, signal transducer and activator of transcript 3 and 5; TCPTP, T cell protein tyrosine phosphatase. The ‘?’ indicates a pathway for which there is only limited published support.

Figure 1 highlights the complexity of the LepRb signalling complex and downstream transcriptional effectors activated by leptin-LepRb binding. The leptin receptor lacks intrinsic tyrosine kinase catalytic activity. The tyrosine kinase Janus kinase 2 (JAK2) becomes phosphorylated following leptin-LepRb binding. Activated JAKs then phosphorylate phosphotyrosine residues on the intracellular domain of the LepRb. The LepRb contains three highly conserved intracellular tyrosine residues (tyrosines 985, 1077, and 1138). These activated phosphotyrosines serve as binding sites for signalling molecules, which themselves then become phosphorylated by the JAKs [31]. The canonical transcriptional effector of leptin action is the Janus kinase 2 (JAK2)/signal transducer and activator of transcription 3 (STAT3) pathway, which is absolutely critical for leptin’s effects on body weight and energy homeostasis, but as we and others have shown, not reproduction [32,33]. In the absence of LepRb-STAT3 signalling, leptin is unable to exert its anti-obesity effects, but is still able to exert its permissive fertility effects. Other signalling pathways are therefore involved in mediating leptin’s effects on the network of afferent inputs to the gonadotrophin-releasing hormone neurons governing fertility (such as AgRP/NPY neurons). Nevertheless, it remains very common to validate animal models of ‘leptin resistance’ simply by showing impaired STAT3 signalling, even when fertility outcomes are being considered (e.g., [23,34]).

Leptin-LepRb binding can also activate other intracellular signalling pathways, including the JAK2/STAT5 pathway, the extracellular signal-regulated kinase (ERK) pathway, and the insulin receptor substrate (IRS)-phosphoinositide 3-kinase (PI3K)/Akt pathway, which diverges to separate downstream signalling and effector molecules including forkhead box protein O1 (FOXO1), mammalian target of rapamycin (mTOR), and S6 (as reviewed in [31,35,36]). Additionally, leptin has been shown to signal through a relatively little-studied adenosine monophosphate-activated protein kinase (AMPK) and CREB-regulated transcription coactivator (CRTC) pathway to modulate energy balance and reproductive function [37]. Evidence for direct promoter binding and transcriptional activity of these different leptin signalling molecules on target genes relevant to metabolic and/or reproductive function can be seen in Table 2. Untangling the effects of leptin signalling through these different pathways, and how the sensitivities of the pathways change in response to hyperleptinemia, has become a topic of particular relevance to the multiple functions of leptin.

### 3.1. STAT3 and STAT5

The JAK/STAT3 pathway is the most extensively studied LepRb signalling pathway. STAT3 and STAT5 are recruited to tyrosine 1138 (and also tyrosine 1077 in the case of STAT5) on the LepRb where they are phosphorylated, following which they translocate as dimers to the nucleus where they act as transcription factors, modulating expression of target genes [31] (Figure 1). Several knockout mouse models have been developed to explore the role(s) of STAT3 and STAT5 signalling in leptin regulation of energy homeostasis and fertility (see Table 1). STAT3 is known to directly regulate transcription of several genes including *Agrp*, *Npy*, *Pomc*, *Socs3*, and *Trh* (see Table 2 for details). LepRb-STAT3 signalling is needed for the transcriptional activation of POMC-derived neuropeptides, including alpha-melanocyte stimulating hormone (αMSH) [32], which is a potent anorexigenic signal required for energy homeostasis.

As mentioned previously, disrupting LepRb-STAT3 signalling in mice causes hyperphagia and obesity, but not infertility [32]. However, in this model, STAT3 itself remains intact, and it is simply LepRb activation of STAT3 that is disrupted by substituting tyrosine 1138 of the LepRb, which mediates STAT3 activation, with serine. In a subsequent study, completely ablating STAT3 from all brain cells in mice was shown to completely recapitulate the obesity, diabetes, infertility, growth retardation, and thermal dysregulation observed in *db/db* mice [38]. In another study in female rats, hypothalamic injection of a phosphopeptide inhibitor of STAT3 activation blocked the mid-cycle surge of luteinizing hormone (LH) that drives ovulation in females [39]. These data suggest STAT3 signalling does play a critical role in the regulation of both metabolic and reproductive function. Given the previous finding that LepRb-mediated STAT3 signalling only appears to be critically required for the regulation of metabolic function [32], it seems other activators of STAT3 signalling in non-LepRb cells must be able to compensate for and thus safeguard reproductive function in the absence of LepRb-STAT3 signalling. Interestingly, we [33] and others [40] have shown that deleting STAT3 from all LepRb-expressing cells results in obesity but uncompromised fertility, suggesting that STAT3 activation in non-LepRb cells is sufficient to protect reproductive function. Such potential non-leptin mediators of STAT3 signalling include insulin [41,42], which appears to be able to compensate for the absence of leptin signalling in other fertility studies [43,44], and oestrogens, which are able to act via STAT3 (discussed later).

One closely-related pathway that could potentially be used by leptin to regulate reproductive function is STAT5, which is phosphorylated at tyrosine 1138 and to a lesser extent tyrosine 1077 of the LepRb. Global knockout of STAT5 results in increased food intake and altered regulation of energy expenditure [45], but the effect is milder than that of STAT3 knockout. Furthermore, since STAT5 signalling is activated by other hormones known to increase body weight including prolactin [46,47] and growth hormone [48], the relevance of this finding to leptin signalling requires further investigation. To investigate whether LepRb-STAT5 signalling is required to maintain normal control of bodyweight and fertility, we generated mice exhibiting a LepRb-specific STAT5 knockout [33]. In the absence of LepRb-mediated STAT5 signalling, bodyweight and fertility were not compromised. Leptin therefore exerts its permissive effects on fertility signalling via pathways other than STAT3 and STAT5. However, another paper showed that mutation of tyrosine 1077 on the LepRb led to impairments in oestrous cycling [49].

### 3.2. PI3K/Akt, FOX01 and mTOR/S6

Leptin also recruits the PI3K/Akt signalling pathway, best known as an insulin receptor signalling pathway, to exert some of its metabolic and reproductive effects [50]. Src homology-2 B adaptor protein 1 (SH2B1), an adaptor protein that binds to protein tyrosine kinases such as JAK2 and insulin receptor, recruits insulin receptor substrates (IRS) to the LepRb, causing increased JAK2 kinase activity [51]. IRS proteins then bind to and activate PI3K subunits, leading to PI3K activation and the accumulation of phosphatidylinositol 3,4,5-triphosphate (PIP3). This leads to the activation of 3-phosphoinositide-dependent protein kinase 1 (PDK1) and Akt. Pharmacological inhibition of PI3K inhibits leptin-induced anorexia in rats [52], and POMC-specific deletion of PI3K signalling in murine POMC neurons impairs leptin and insulin’s ability to modulate POMC neuronal firing, but does not affect body weight [53]. Male and female mice exhibiting LepRb-specific deletion of PI3K-p110α or -p110α and -p110β catalytic subunits (which are the subunits required for the acute anorexigenic effects of insulin and leptin [54]) exhibit a lean phenotype, and the female mice also exhibit delayed pubertal development and progressive subfertility [55]. These data demonstrate leptin exerts some of its reproductive control via PI3K signalling, whereas metabolic functions exhibited enhanced leptin sensitivity due to increased leptin-induced STAT3 signalling. Interestingly, the delayed pubertal development observed in female mice exhibiting LepRb-specific PI3K p110α deletion was partially corrected by early postnatal overnutrition, which suggests the lean phenotype and consequent low leptin levels were underlying some of the delayed pubertal development. Nevertheless, the results support the idea that metabolic and reproductive control rely on distinct leptin signalling pathways. Leptin treatment was unable to advance pubertal timing in the absence of PI3K p110α signalling [55], suggesting leptin signalling via PI3K may play a critical role in the regulation of puberty onset and fertility.

PI3K/Akt signalling diverges to multiple downstream effectors, including FOX01 and mTOR/S6 (Figure 1), and which ones are involved in mediating leptin’s actions, particularly with respect to reproduction, remains unclear. Activation of Akt inhibits the activation (dephosphorylation) of the transcription factor FOX01. Activated FOX01 stimulates *Agrp* and *Npy* transcription and suppresses *Pomc* transcription [56,57,58]. These effects are the opposite of those of activated STAT3, which inhibits *Agrp* and *Npy* transcription, but the overall effect of leptin on these genes via the two pathways is similar since PI3K/Akt signalling inhibits FOX01 activation (hence, deletion of PI3K or FOX01 from POMC neurons results in opposite effects [59,60]). The role of FOX01 in modulating reproductive function remains unknown. 

Leptin is also able to stimulate the phosphorylation of p70 S6 kinase (S6K) via the PI3K/Akt pathway and the serine-threonine kinase mTOR. Mice lacking S6K do not respond to the anorectic actions of leptin [61,62]. There is evidence for stimulation of reproduction by the mTOR pathway. Activation of mTOR by l-leucine stimulated LH secretion in pubertal and food-restricted female rats, and blockade of mTOR signalling with rapamycin reduced reproductive hormone and kisspeptin gene expression levels and delayed normal and leptin-induced female puberty [63]. Activation of S6K mediates the phosphorylation of the 40S ribosomal protein subunit S6 [38], which then regulates protein translation, but the same outcome can be achieved in an mTOR-independent manner via the ERK1/2 signalling cascade [64] (described below).

### 3.3. ERK1/2

ERK1/2, members of the mitogen-activated protein kinase (MAPK) superfamily, form part of a signalling cascade downstream of the tyrosine 985 binding site on the LepRb [65] that mediates the activation of downstream signalling molecules including c-Fos [66], cAMP response element-binding protein (CREB) [67], early growth response protein 1 (EGR1) [68] and S6 [69], many of which then become components of gene transcription complexes (Figure 1, summary detail only shown). LepRb modulates the activity of ERK1/2 via JAK2, as blockade of JAK2 inhibits ERK1/2 activation by leptin in a dose dependent manner [70]. The leptin receptor recruits the adaptor protein growth factor receptor-bound protein 2 (Grb2) via another adaptor protein, Src homology-2 domain-containing protein tyrosine phosphatase-2 (SHP2). The inability of leptin to stimulate ERK1/2 in the absence of SHP2 suggests that this protein stimulates leptin activation of ERK1/2 [71], in contrast to the inhibitory actions of the three tyrosine phosphatases discussed in the leptin resistance section below. Other kinases that act as signalling intermediaries in the tiers between SHP2 and ERK1/2 are Raf and MEK [72]. One important nuclear target of ERK1/2 signalling is AP-1; a transcription factor complex composed of Jun and Fos family members that binds to specific control elements in numerous genes that regulate cell differentiation and proliferation [73]. Alternatively, ERK1/2 can converge on the PI3K/Akt/mTOR/S6 pathway by activating p90 ribosomal S6 kinase (RSK), which is able to phosphorylate S6 in a slightly more limited manner than S6K does [64].

Administration of leptin causes a marked increase in immunoreactive ERK1/2 (but not another MAPK family member, p38 MAPK) in the arcuate nucleus [68,70] in a manner that is not dependent on STAT3 or STAT5 presence [33]. Interestingly, all the neurons in which leptin increased ERK1/2 immunoreactivity were POMC positive, with no ERK1/2 activation observed in NPY neurons [68,70]. These data suggest that ERK1/2 mediates leptin action through an effect on POMC neurons [70], but the specific genes targeted are yet to be identified. Pharmacological blockade of hypothalamic ERK1/2 reversed the anorectic and weight-reducing effects of leptin, suggesting that the ERK1/2 pathway can modulate the metabolic actions of leptin [70].

The ERK1/2 pathway may play a role in the regulation of fertility, as neuronal deletion of SHP2 (using a CaMKIIα Cre x *Shp2*-flox mouse line) yielded mice in which only 66% of females were fertile [71]. This potential role of ERK1/2 warrants further investigation.

### 3.4. CRTC

More recently, it has been suggested that leptin also signals through CRTCs, formerly known as transducers of regulated CREB activity (TORC, a name easily confused with the mammalian target of rapamycin complexes mTORC1 and mTORC2). CRTCs are transcriptional coactivators predominantly expressed in the brain [74]. Leptin enhances the dephosphorylation and nuclear translocation of CRTC1 in the arcuate nucleus (Figure 1), where it binds to CREB to activate the transcription of several tissue-specific CREB-regulated genes including cocaine and amphetamine regulated transcript (*Cartpt*) and kisspeptin (*Kiss1*) [74], both of which modulate energy balance and reproductive function [74,75]. The association between Crtc1 signalling and *Kiss1* is the only example to date of a gene primarily concerned with reproduction that is modulated by a LepRb signalling pathway. The signalling events that link CRTC1 to the LepRb remain uncertain, but may involve inhibition of AMPK activity by leptin [76] which then alleviates the inhibitory effect of AMPK on CRTC activation [77]. 

Using a CRTC1 knockout mouse line, two independent groups observed that a whole-body deficiency of CRTC1 caused a severe impairment in fertility, with no surviving offspring obtained from knockout male or female mice when paired with wildtype counterparts [75,78]. Additionally, knockout females displayed abnormal uterine morphology and low circulating LH level, a key hormonal regulator of gonadal function and ovulation [75,78]. However, CRTC1 knockout mice were only moderately obese, potentially reflective of compensation from other leptin signalling pathways including related CRTC family members [75,78]. Quantitative polymerase chain reaction and in situ hybridization analysis confirmed downregulation of *Cartpt* and *Kiss1* in CRTC1 knockout mice [75]. Contrary to these findings, a subsequent study employed the same insertional mutagenesis technique to create a CRTC1 mouse line and reported no signs of infertility [79]. Their CRTC1 knockout mice did develop an obese phenotype on a normal chow diet, emphasising the importance of CRTC1 for the control of energy balance. These contrasting findings emphasize the need for further studies to evaluate the contribution of CRTC1 to leptin’s effects on the reproductive neuroendocrine axis [80]. 

### 3.5. Interactions between Leptin and Oestrogen Receptor (ER) Signalling

Oestrogens can activate signalling pathways associated with the LepRb; in endothelial cells, oestradiol rapidly phosphorylates STAT3 and STAT5 [81], and rapid (within 30 min), leptin-independent STAT3 phosphorylation was also observed in oestradiol-treated mice [82]. These authors also showed that the oestrogen-induced decrease in body weight in female mice was prevented by brain-wide STAT3 knockout. In contrast, STAT3 in LepRb cells is not required for oestrogen-induced body weight suppression in female mice [83], suggesting that oestradiol can exert this effect via non LepRb cells. Surprisingly, leptin may be able to activate ERα, at least in vitro, via LepRb signalling pathways such as MAPK [84] and STAT pathways. The ERα promoter contains STAT3 [85] and STAT5 [86] response elements. In BG-1 ovarian cancer cells, leptin-induced ER activation was blocked by STAT3 inhibition [85].

## 4. The Pathogenesis of Leptin Resistance

Obese individuals generally exhibit circulating leptin concentrations in proportion to their increased adiposity [87], yet they are refractory to its effects, even in response to a pharmacological dose [88]. At least three underlying defects contribute to the inability of leptin to exert its effects on hypothalamic LepRb-expressing neurons; these are decreased transport across the blood–brain barrier to hypothalamic sites of action, reduced trafficking and recycling of the LepRb, and reduced activation of leptin-LepRb-induced intracellular signalling [36,88]. The mechanisms underpinning reduced intracellular LepRb signalling are discussed below. Like many other homeostatic systems, leptin signalling is regulated in a negative feedback manner. In response to excessive leptin-LepRb binding, several signalling pathways are activated that attenuate leptin-targeted signalling cascades (Figure 1). 

### 4.1. SOCS3

Suppressor of cytokine signalling 3 (SOCS3) acts as a feedback inhibitor of leptin signalling via the JAK/STAT pathway, whereby increased STAT3 activation in response to leptin signalling induces SOCS3 expression, which in turn inhibits JAK2 phosphorylation [18]. In addition to this inhibition of JAK2, SOCS3 has also been shown to bind tyrosine 985 on the LepRb and consequently mediate the inhibition of STAT3 activation [89] (Figure 1). Mutation of the STAT3-recruiting tyrosine 1138 on the LepRb prevented SOCS3 feedback inhibition of leptin signalling [90], while overexpression of a constitutively active version of STAT3 in POMC neurons elevated *Socs3* expression and led to obesity [91]. Not surprisingly, SOCS3 expression is elevated in leptin resistant obese mice due to their hyperleptinemia, whereas related SOCS family members are not induced by leptin [92]. Mice exhibiting haploinsufficiency of *Socs3* are partially protected from both obesity and leptin resistance in response to high-fat feeding [93]. These data, among others [94,95], importantly demonstrate that negative regulation of leptin signalling via SOCS3 contributes to the metabolic dysfunction associated with obesity-related leptin resistance.

To investigate whether upregulated SOCS3 also plays a role in high calorie diet (HCD)-induced infertility, our group generated mice exhibiting SOCS3 knockout from all forebrain neurons and monitored reproductive and metabolic parameters in response to HCD feeding. Interestingly, male and female neuron-specific SOCS3 knockout mice showed different levels of protection from leptin resistance in response to HCD-feeding, whereby the male knockout vs. control mice were almost entirely protected from HCD-induced obesity, whereas the female knockout vs. control mice exhibited very limited protection from HCD-induced obesity [23]. Furthermore, while HCD-fed male mice remained completely fertile, the female control mice developed HCD-induced infertility and this was prevented for over a month by SOCS3 knockout. This improvement in fertility in the HCD-fed neuron-specific SOCS3 knockout females did not appear to be secondary to any metabolic improvements due to the relatively limited effects of SOCS3 knockout on female metabolic function [23], further supporting the hypothesis that leptin (and the emergence of leptin resistance) can differentially impact metabolic and reproductive function. Other negative regulators of leptin signalling are also involved, and over time may compensate for the lack of SOCS3 signalling. 

### 4.2. PTP1B

Another important negative regulator of leptin signalling is protein tyrosine phosphatase 1B (PTP1B), which dephosphorylates JAK2 to suppress downstream phosphorylation of STAT3 [96] (Figure 1). PTP1B may also dephosphorylate IRS 1 [97]. PTP1B is elevated in obesity, and both whole body and brain-specific PTP1B knockout mice exhibit improved leptin sensitivity and partial resistance to diet-induced obesity [96,98]. We were therefore interested in determining whether PTP1B activation also contributes to HCD-related infertility. To this end, we investigated whether neuron-specific PTP1B deletion could protect female mice from developing HCD-related infertility. Despite showing partial protection from diet-induced obesity, which is consistent with previous findings [96,99,100], the neuron-specific PTP1B knockout mice showed no improvements in fertility (Ancel et al., under review).

### 4.3. PTPe

Receptor-type protein tyrosine phosphatase epsilon (RPTPe) and its cytosolic PTPe variant are other negative regulators of hypothalamic leptin signalling. Leptin-induced phosphorylation of PTPe causes it to dephosphorylate JAK2 [101] (Figure 1), forming a negative feedback pathway of leptin signalling similar to that created by SOCS3. It may also inhibit MAPK signalling [102]. Deletion of PTPe variants in mice reduces female obesity and improves blood glucose control [101]. Its effects on diet-induced infertility are unknown, but the female-specific protection from obesity makes it of interest in this regard since male mice are much less prone to diet-induced infertility [23].

### 4.4. TCPTP

T cell protein tyrosine phosphatase (TCPTP) can likewise attenuate leptin signalling by dephosphorylating STAT3 (Figure 1), which is consistent with it serving as a negative feedback regulator of leptin signalling. TCPTP expression is regulated by metabolic state, such that fasting increases and feeding suppresses its expression via a glucocorticoid-mediated mechanism [103]. Consistent with it playing a role in the pathophysiology of hyperleptinemia-induced leptin resistance, TCPTP expression is elevated in the hypothalami of obese mice, and it is also elevated in response to leptin administration [104]. As expected, neuronal TCPTP-deficient mice exhibit enhanced leptin sensitivity, as assessed by a reduction in food intake and body weight after leptin administration, as well as increased leptin-induced hypothalamic pSTAT3 signalling [104]. Similarly, mice exhibiting AgRP/NPY-specific TCPTP deficiency are resistant to diet-induced obesity and exhibit increased energy expenditure [103]. Interestingly, neither SOCS3 nor PTP1B proteins show any changes during fasting or re-feeding in either chow-fed or high-fat-fed obese mice, whereas TCPTP protein levels and mRNA expression are significantly reduced in chow-fed fasted mice that have been re-fed for 4 h, which is not observed in the corresponding high-fat-fed mice [103]. Therefore, the TCPTP ‘feed-fast switch’ appears to be abrogated in obesity, which could promote elevated TCPTP expression and subsequent STAT3 dephosphorylation, even in the fed state, thereby further promoting obesity. It remains unknown whether TCPTP plays a role in mediating obesity-related infertility. 

### 4.5. PTEN

Phosphatase and tensin homolog deleted on chromosome 10 (PTEN) acts primarily by dephosphorylating PIP3 into PIP2 and thereby inhibiting PI3K signalling in response to leptin or insulin receptor activation [105] (Figure 1). Mice with LepRb-specific knockout of PTEN exhibit a lean phenotype that appears to be primarily due to increased energy expenditure [106].

## 5. Summary and Areas for Future Focus

In the hypothalamus, LepRb signalling plays a critical role in the regulation and integration of metabolic and reproductive function. The neuronal pathways whereby leptin-LepRb signalling contribute to both metabolic and reproductive control are becoming increasingly well characterized [24], yet many unresolved questions remain when it comes to understanding how hyperleptinemia-induced leptin resistance differentially impacts metabolic and reproductive function. Completely deleting or rescuing LepRbs from discrete neuronal populations is a straightforward and effective way to characterize whether leptin signalling via the targeted neuronal population(s) plays a critical or sufficient functional role, respectively, but it is an ‘all or nothing’ approach that does not model physiologically-relevant conditions such as leptin resistance, in which leptin signalling falls somewhere between ‘all’ and ‘nothing’. Understanding how changes in leptin sensitivity differentially affect leptin-LepRb signalling pathways and their respective transcriptional targets (i.e., metabolically-relevant vs. reproductively-relevant genes) within the same LepRb-expressing neuron(s) is much more experimentally challenging. 

Nevertheless, as we have reviewed, some headway has been made in trying to understand how leptin exerts its discrete effects on metabolic vs. reproductive function, and how the development of leptin resistance differentially affects metabolic vs. reproductive leptin-target genes. The apparent total lack of requirement for leptin-induced STAT3 for control of fertility in mice remains surprising and somewhat controversial, although the contradictory findings may be explained by the fact that the data highlighting the critical role of STAT3 in the control of fertility were based in manipulations that included non-LepRb cells. Limited data implicates ERK1/2, PI3k-Akt, and CRTC1 pathways as having a role in modulating reproductive function, but it remains to be identified whether there is a signalling pathway that is absolutely required for fertility. The sensitivities of the different intracellular signalling pathways activated by leptin-LepRb binding do not appear to change uniformly in the face of hyperleptinemia-induced leptin resistance, perhaps because the signalling pathways are constrained by different negative regulators. The primary known negative regulators of leptin signalling (SOCS3, PTP1B, PTPe and TCPTP) mostly target STAT3 signalling, which impacts metabolic function more critically than reproductive function [32], for example. To date, much less is known about the negative regulation of non-STAT3 intracellular signalling pathways involved in mediating leptin’s effects. While protein tyrosine phosphatases (particularly PTEN) are known to modulate non-STAT signalling, there is yet to be an inhibitor identified that completely protects against diet-induced infertility. 

Furthermore, many unknowns remain when it comes to understanding the specific genes that are targeted by different leptin-activated intracellular signalling pathways, particularly those that impact on reproduction. Most of the efforts to identify central targets of leptin signalling have focused on genes that code for the metabolically-relevant POMC, NPY and AgRP peptides, although the reproductively-important *Kiss1* has also been identified as a CRTC1 target gene. It should be noted that the arcuate neurons that express these genes make up only a small proportion of the total number of LepRb cells in the brain [107]. Therefore, considerably more work will be required to discover the full extent of the cells and genes targeted by specific leptin-LepRb signalling pathways.

**Table 1:** Leptin signalling molecule knockout studies focusing on metabolic and reproductive function. See Figure 1 legend for abbreviations of signalling molecules.

Signalling Molecule Deleted	Cell Type Deleted from	Description of Findings	Reference
STAT3	LepRb cells	Obesity but not delayed puberty or infertility. No effect on oestrogen-induced weight loss.	[32,33,40,83]
	Brain cells	Obesity and infertility.	[38]
	POMC neurons	Mild obesity, defects in compensatory refeeding and decreased *Pomc* mRNA expression.	[108]
	AgRP neurons	Modest weight gain, increased *Npy*, decreased *Socs3* mRNA.	[109]
STAT5	Global	Increased food intake and altered energy expenditure.	[45]
	LepRb cells	No effect on bodyweight, puberty timing or fertility.	[33]
PI3K	POMC neurons	Reduced sensitivity to leptin’s anorectic effects.	[59]
PI3K catalytic subunits, p110α and p110β	POMC or AgRP neurons	POMC p110β (but not p110α KO) KO: leptin resistance, obesity, loss of insulin- and leptin-stimulated neuronal firing. AgRP p110β (but not p110α KO) KO: increased leptin sensitivity, resistance to diet-induced obesity. POMC PI3K KO mice: disrupted leptin and insulin-induced POMC neuronal firing.	[110] [53]
	Kisspeptin neurons	Reduced kisspeptin cell number in AVPV (females) and arcuate nucleus (males); reduced female fertility.	[111]
SH2B1	Global	Hyperphagia, obesity. Resolved by neuronal SB2B1 rescue.	[112,113]
	LepRb cells	Obesity, insulin resistance.	[114]
PTEN	LepRb cells	Increased PI3K activity, reduced adiposity.	[115]
	POMC neurons	Leptin resistance, obesity, reduced POMC firing.	[106]
FOX01	POMC neurons	Increased sensitivity to leptin’s anorectic effects.	[60]
	AgRP neurons	Reduced food intake, increased glucose and leptin sensitivity.	[116]
S6 kinase	Global	Leptin insensitivity. Protection against obesity, increases insulin sensitivity.	[61] [62]
	POMC or AgRP neurons	Impaired glucose homeostasis and altered POMC and AgRP neuronal excitability, no effect on food intake or bodyweight.	[117]
SHP2	Brain neurons	Obesity, reduced leptin-induced pERK1/2 (pSTAT3 was preserved). Reproductive impairment.	[71]
AMPKα	POMC or AgRP neurons	POMC KO: obesity but remained sensitive to leptin. AgRP KO: age-dependent lean phenotype.	[118]
	Kisspeptin neurons	AMPKα2-KO: fasting-induced disruption of oestrous cycles prevented. AMPKα1 KO: subnutrition-induced puberty delay prevented.	[119,120]
CRTC1	Global	Obesity and infertility (Altarejos) Obesity but normal fertility (Breuillaud).	[37] [79]
SOCS3	Global	+/− mice have reduced diet-induced obesity and leptin resistance.	[93]
	Brain cells	Protection from diet-induced obesity.	[94]
	Brain neurons	Protection from diet-induced obesity (most evident in males) and leptin resistance; delayed the onset of diet-induced infertility in females.	[23]
	LepRb cells	Reduced food intake after fasting; reduced *Agrp* and *Npy* mRNA.	[121]
	POMC neurons	Improved leptin sensitivity and glucose homeostasis.	[95]
PTP1b	Global	Resistance to diabetes and diet-induced obesity.	[98]
	Brain cells	Resistance to diet-induced obesity, hypersensitive to leptin.	[96,99]
	Brain neurons	Resistance to diet-induced obesity, not diet-induced female infertility.	[122]
	LepRb cells	Resistance to diet-induced obesity, hypersensitive to leptin.	[100]
	POMC neurons	Resistance to diet-induced obesity, hypersensitive to leptin.	[123]
PTPe	Global	Reduced female obesity and improved blood glucose control.	[101]
TCPTP	Brain cells	Enhanced leptin sensitivity, reduced leptin-induced food intake.	[104]
	AgRP neurons	Resistant to diet-induced obesity, increased energy expenditure.	[103]
PTEN	LepRb cells	Lean phenotype due to increased energy expenditure.	[106]

**Table 2:** Evidence for direct promotor binding and transcriptional activity of leptin signalling molecules on target genes relevant to metabolic and reproductive function. See Figure 1 legend for abbreviations of signalling molecules.

Signalling Pathway	Target Gene	Description of Findings	Reference
STAT3	*Npy*	Leptin actions via the 221-bp region of the *Npy* promotor, which possesses two putative STAT3 binding sites.	[124]
	*Agrp*	STAT binding sites upstream of *Agrp* (but not STAT3 itself) are required for fasting-induced *Agrp* transcription.	[124,125]
	*Pomc*	Leptin and STAT3 increased *Pomc* promoter activity. A 30-bp promoter element is required for leptin regulation.	[58,126]
	TRH	STAT3-response elements identified in *Trh* promoter.	[127]
	*Socs3*	STAT3-bindis to the *Socs3* promoter.	[127]
	*ERα*	*ERα* promoter contains a STAT3 response element.	[85]
FOX01 and STAT3	*Agrp* and *Pomc*	Leptin-induced phospho-STAT3 activates the *POMC* promoter via an SP1-binding site which overlaps with a FOX01-binding element. FOXO1 binds to STAT3 and prevents it from interacting with the promoter. FOXO1 and STAT3 exert opposing actions on *Agrp* and *Pomc* expression. FOXO1 activates *Agrp* and inhibitis *Pomc*.	[56,57,58]
FOX01	*NPY, AGRP* and *Pomc*	FOXO1 binds upstream of the *NPY* coding region and increases *NPY* promoter activity. Foxo1-mediated *NPY* transcription is negatively regulated by leptin, insulin and PI3K/Akt signalling. Foxo1 increases *AGRP* promoter activity but not *Pomc* promoter activity.	[58]
AMPK-CRTC1	*Cartpt* and *Kiss1*	*Cartpt* and *Kiss1* promoters contain CREB binding sites. Leptin recruits CRTC1 to *Cart* and *Kiss1* promoters.	[37]

## Data Availability

Not applicable as this review article does not include original data.
